# Electric-field induced modulation of amorphous protein aggregates: polarization, deformation, and reorientation

**DOI:** 10.1038/s41598-022-06995-x

**Published:** 2022-02-23

**Authors:** Kyongok Kang, Florian Platten

**Affiliations:** 1 Forschungszentrum Jülich, Institute of Biological Information Processing IBI-4, Biomacromolecular Systems and Processes, Jülich, Germany; 2 Condensed Matter Physics Laboratory, Heinrich Heine University, Düsseldorf, Germany

**Keywords:** Biophysical chemistry, Protein aggregation

## Abstract

Proteins in their native state are only marginally stable and tend to aggregate. However, protein misfolding and condensation are often associated with undesired processes, such as pathogenesis, or unwanted properties, such as reduced biological activity, immunogenicity, or uncontrolled materials properties. Therefore, controlling protein aggregation is very important, but still a major challenge in various fields, including medicine, pharmacology, food processing, and materials science. Here, flexible, amorphous, micron-sized protein aggregates composed of lysozyme molecules reduced by dithiothreitol are used as a model system. The preformed amorphous protein aggregates are exposed to a weak alternating current electric field. Their field response is followed in situ by time-resolved polarized optical microscopy, revealing field-induced deformation, reorientation and enhanced polarization as well as the disintegration of large clusters of aggregates. Small-angle dynamic light scattering was applied to probe the collective microscopic dynamics of amorphous aggregate suspensions. Field-enhanced local oscillations of the intensity auto-correlation function are observed and related to two distinguishable elastic moduli. Our results validate the prospects of electric fields for controlling protein aggregation processes.

## Introduction

Proteins are only marginally stable against unfolding and aggregation. Given appropriate conditions, they readily lose their native structure as well as their biological function and stick together into insoluble assemblies^1–4^. These condensed states can be ordered or amorphous. Protein aggregation is vitally important in various disciplines, ranging from medicine^5^ over pharmacology^6^ and food processing^7^ to materials science^8^. Medically, the deposition of insoluble protein aggregates (PAs) is associated with several disorders, such as Alzheimer’s disease and type II diabetes mellitus^9^. In protein drug products, high-concentration formulations are often required, e.g., for subcutaneous administration of a therapeutic dose; however, these solutions are prone to aggregate, posing the risk of immunogenicity^10^. In food protein systems, a solid-like texture can be achieved by heat-induced aggregation^11^. Moreover, functional materials composed of proteins or protein aggregates are used in nature and in applications, e.g., as catalytic scaffolds and as a drug depot, respectively^12^.

As a consequence, attempts to control the aggregation process or to manipulate mature protein aggregates are required, but challenging. Avoiding aggregation plays an important role in protein drug development^13^ and in therapeutic approaches towards misfolding diseases^14^. Inhibition strategies include the stabilization of the native state by additives, like glycerol^15^, and the suppression of aggregation by excipients^16^, nanoparticles^17^, and small-molecule inhibitors, like polyphenols^18^. Molecular chaperones, such as heat shock proteins, represent another example as they not only prevent misfolding, but also regulate protein aggregation^19–22^. The chaperone activity can be facilitated by conformational changes like the rotation of domains^23^ and by hydration properties. Although aggregation is unwanted in the previous examples, it has proven beneficial in food and materials science^24^ in order to tailor product properties. Amorphous aggregates and amyloid fibrils can form at high temperatures or in the presence of reducing agents, such as dithiothreitol (DTT)^25–28^. Moreover, in some works, external stimuli have been used to alter the aggregation process and aggregate properties. For example, agitation and shear flow can induce and accelerate protein aggregation^29^. While electric fields have been used a lot to tune the collective behavior of colloidal suspensions^30,31^, less is known about electric-field effects on protein condensation. Pulsed fields have been applied to pasteurize food protein products^32^. Oscillating electric fields can stabilze or destabilize protein conformation^33,34^. Electric fields can disrupt amyloid fibrils^35^ and have even been considered as a potential non-invasive therapy against condensation diseases^36,37^. Nevertheless, the systematic knowledge about electric-field effects on protein aggregates is still very limited.

In the present work, the response of amorphous protein aggregates to low strength alternating current (AC) electric fields is explored in-situ. Lysozyme, an enzyme that catalyzes the hydrolysis of components embedded in many bacterial cell walls, is one of the most widely studied proteins in the context of protein (mis)folding and condensation^38–44^. Moreover, the effects of an external electric field on the relaxation dynamics of molecular lysozyme solutions with antagonistic salt have recently been examined^45^. Therefore lysozyme serves as a well-suited model protein here. In the presence of DTT, lysozyme readily forms micron-sized, highly flexible, amorphous morphologies^25^. Here, the field response of such preformed aggregates is analyzed in situ by time-resolved polarized optical microscopy and small-angle dynamic light scattering (DLS). This work thus aims at further exploring the potential of electric fields as a means to manipulate protein aggregates.

## Results

First, the features of the amorphous protein aggregates (PAs) formed at various total protein concentrations *c* are described (in the absence of an electric field). Second, time-resolved optical microscopy reveals elastic deformations, field-enhanced polarization and reorientations of protein aggregates. The effects on samples with different *c* are subsequently discussed: intermediate ($c=1~\mathrm {mg/ml}$), high ($c=4~\mathrm {mg/ml}$) and low ($c=0.4~\mathrm {mg/ml}$). Third, field-induced modulations of the collective microscopic dynamics of PA suspensions are investigated by DLS for two samples ($c=0.1~\mathrm {mg/ml}$ and $c=0.4~\mathrm {mg/ml}$) and related to two distinguishable elastic moduli.

### Features of the amorphous protein aggregates in the absence of an electric field

**Figure 1:**
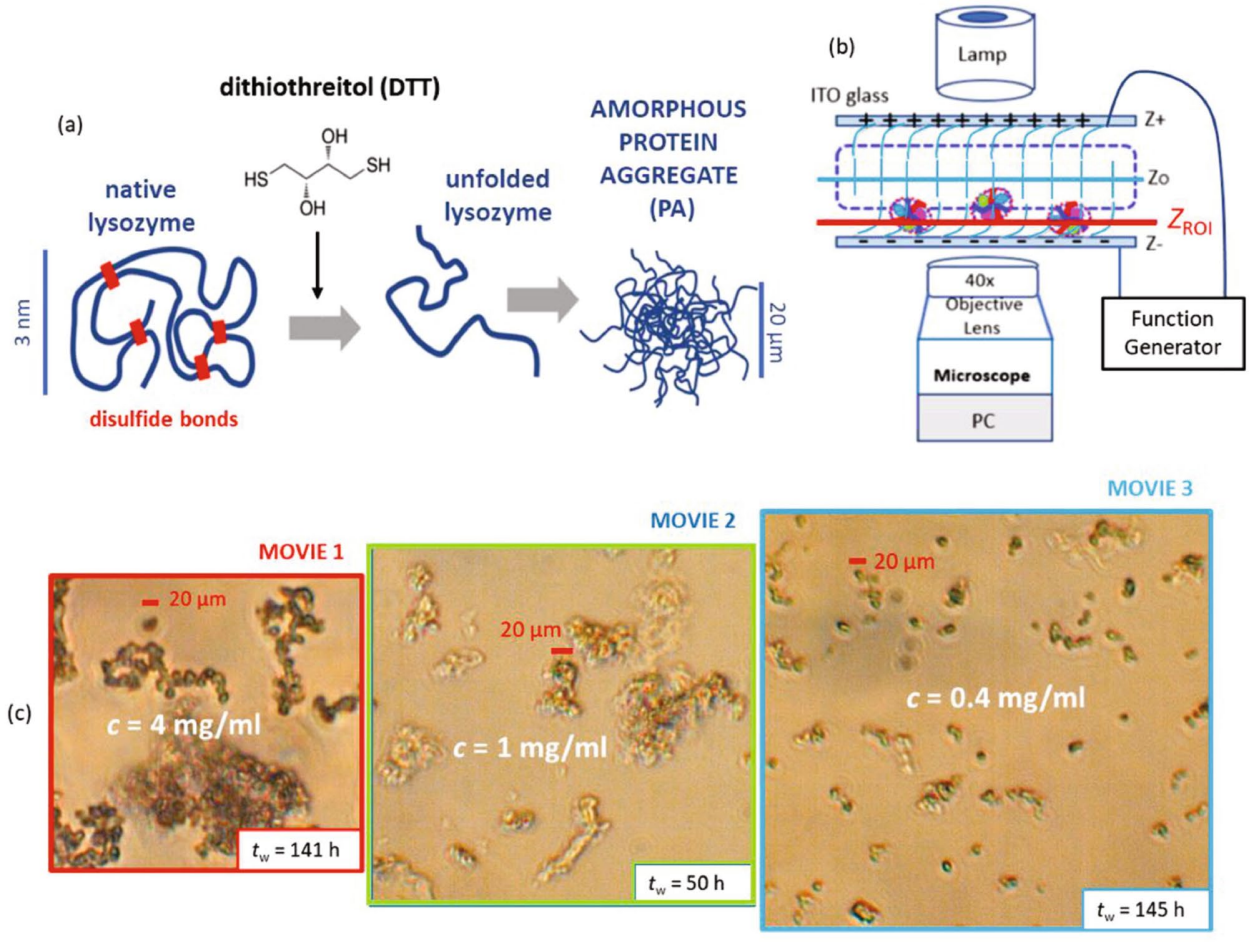
(**a**) Schematic illustration of the formation of amorphous protein aggregates: four disulfide bridges (red bars) stabilize the native conformation of the lysozyme molecule (polypeptide backbone shown as blue line). Upon addition of the reducing agent DTT, these bonds break and the protein unfolds, attaining a collapsed misfolded, random coil-like conformation. The reduced proteins largely assemble into mesoscopic, flexible amorphous structures (blue conglomerate). The response of these aggregates to electric fields is explored in the present work. (**b**) The sample solution is filled into an in-situ electric-field optical cell. The cell is composed of two separated ITO glasses, which are connected to a function generator. It can be mounted onto an inverted optical microscope (as schematically shown) or onto a dynamic light scattering set-up (cf. Fig. 7). The *z*-plane, $z_\text {ROI}$, (indicated by the red line) is the region of interest and taken slightly above the bottom ITO plate. (**c**) Optical morphologies of protein aggregates (PAs) in the absence of an electric field taken a long time $t_\text {w}$ after sample preparation, such that most reduced protein monomers have been integrated into the aggregates. The samples differ in their total protein concentrations *c* and hence also in their typical aggregate size. Movies of the three systems (MOVIE 1, 2, 3) exposed to electric fields are provided as supplementary information.

Figure 1 introduces the system under investigation: amorphous protein aggregates. The figure illustrates (a) the formation of the amorphous protein aggregates, (b) the in-situ electric cell used for microscopic and light-scattering investigations, and (c) typical optical morphologies of the amorphous protein aggregates. In this section, features of the protein aggregates in the absence of an electric field are described. In the following sections, their responses to electric fields are investigated.

Upon addition of DTT (see Fig. 1a), a disulfide-reducing agent, lysozyme is likely to denature and attain a collapsed misfolded, random coil-like structure^46,47^. At near-neutral pH, denatured lysozyme molecules can assemble into particle-like, non-fibrillar aggregates^48,49^. The aggregation process is expected to be largely driven by the hydrophobic effect. If the disulfide-bond reduction is incomplete, the aggregates are also likely to be affected by the formation of scrambled intra- and intermolecular disulfide bonds^25^. According to a recent protocol^25^, DTT-reduced lysozyme is prone to form large-scale amorphous aggregates at neutral pH. After an incubation time of $4~\mathrm {h}$, almost all protein molecules have been integrated into aggregates and only very few remain soluble, as inferred from UV absorbance measurements of the supernatant^25^. The aggregates were characterized, e.g., by scanning electron microscopy as well as intrinsic and extrinsic fluorescence and found to be highly flexible, non-fibrillar and amorphous with a typical diameter of $0.4~\upmu \mathrm {m}$ and a common size of $30~\upmu \mathrm {m}$^25^. In order to obtain amorphous protein aggregates, an experimental approach similar to that of Ref.^25^ was adopted in this work.

Here, protein solutions containing $20~\mathrm {mM}$ DTT were prepared at pH 7.0 and at an ionic strength of $0.1~\mathrm {M}$ (due to the sodium phosphate buffer and added NaCl). The solutions differed in their total protein concentrations *c*, resulting in aggregates that vary in size and flexibility. For the highest protein concentration used, $c=4~\mathrm {mg/ml}$, this corresponds to an excess of 71.6 DTT molecules per lysozyme. After preparation, the samples were stored for at least two days at ambient conditions, such that mature aggregates form.

Sample solutions containing the preformed amorphous aggregates were carefully filled inside the in-situ electro-optical cell illustrated in Fig. 1b. It is used for the investigations of the real-space morphology and collective microscopic dynamics by time-resolved optical microscopy and dynamic light scattering, respectively, also in the presence of an electric field^50^. These methods allow us to observe the field-induced changes of the configuration, orientation, polarization, and collective dynamics of protein aggregate suspensions. Note that the plane of interest in the field of view, $z_\text {ROI}$, is located just above the lower indium-tin-oxide (ITO) glass.

The electrodes used here consist of glass coated with a thin ITO layer, which is in direct contact with the protein suspension. The capacity of this thin layer is sufficiently small, so that the applied field strength is not significantly different from the actual field strength within the suspension. Electrode polarization effects, however, can give rise to differences between the applied and actual field strength at low frequencies. For the cell gap width of 1 mm, and for the frequencies used here, such effects are negligibly small^51–53^.

Before exposing the samples to electric fields, the “field-free” optical morphologies of the amorphous aggregates (Fig. 1c) were inspected. In agreement with Ref.^25^, mature, micron-sized amorphous aggregates were observed, indicating that the aggregation process is indeed completed. Nevertheless, for each sample, the time between preparation and observation, $t_\text {w}$, is indicated. Although most protein molecules are likely to be incorporated into the aggregates, as observed previously^25^, the solutions are labelled according to their total monomer concentration *c* for brevity.

The exemplary micrographs of the aggregate suspensions (Fig. 1c) indicate an amorphous aggregate morphology. Note that at a high concentration ($c=4~\mathrm {mg/ml}$), more elongated and extended clusters of aggregates form, while smaller clusters occur at a low concentration ($c=0.4~\mathrm {mg/ml}$). Overall, the aggregate size is found to increase with *c*, as more protein molecules can be incorporated into the aggregates at higher *c*. For instance, the size of the largest aggregate, as inferred from Fig. 1c, is about $300~\upmu \mathrm {m}$, $200~\upmu \mathrm {m}$, and $40~\upmu \mathrm {m}$, for $c=4~\mathrm {mg/ml}$, $1~\mathrm {mg/ml}$, and $0.4~\mathrm {mg/ml}$, respectively. In order to highlight the different aggregate sizes, a larger field of view is provided for systems with a lower *c*.

As discussed in the following sections, the samples were then exposed to an external weak AC electric field and monitored in-situ for typically $4~\mathrm {h}$ by time-resolved optical microscopy ($c=4~\mathrm {mg/ml}$, $1~\mathrm {mg/ml}$, and $0.4~\mathrm {mg/ml}$) or dynamic light scattering ($c=0.4~\mathrm {mg/ml}$ and $0.1~\mathrm {mg/ml}$). Movie data on the field response of the aggregates shown in Fig. 1c is available as supporting information (MOVIE 1, 2, and 3 again with different field of views). The field response of certain protein aggregates (labelled as PA with various subscripts for the different *c*) is examined during the local time span *t*.

Note that only relatively weak electric fields (a few V/mm) are required to affect the amorphous aggregates in comparison to those used to alter protein secondary structure and promote fibril formation^54,55^. The reason for this might be the weak inter-protein interaction within the amorphous aggregates.

### Characteristic features of large amorphous protein aggregates under an AC electric field (formed at $c=1~\mathrm {mg/ml}$ and $c=4~\mathrm {mg/ml}$)

**Figure 2:**
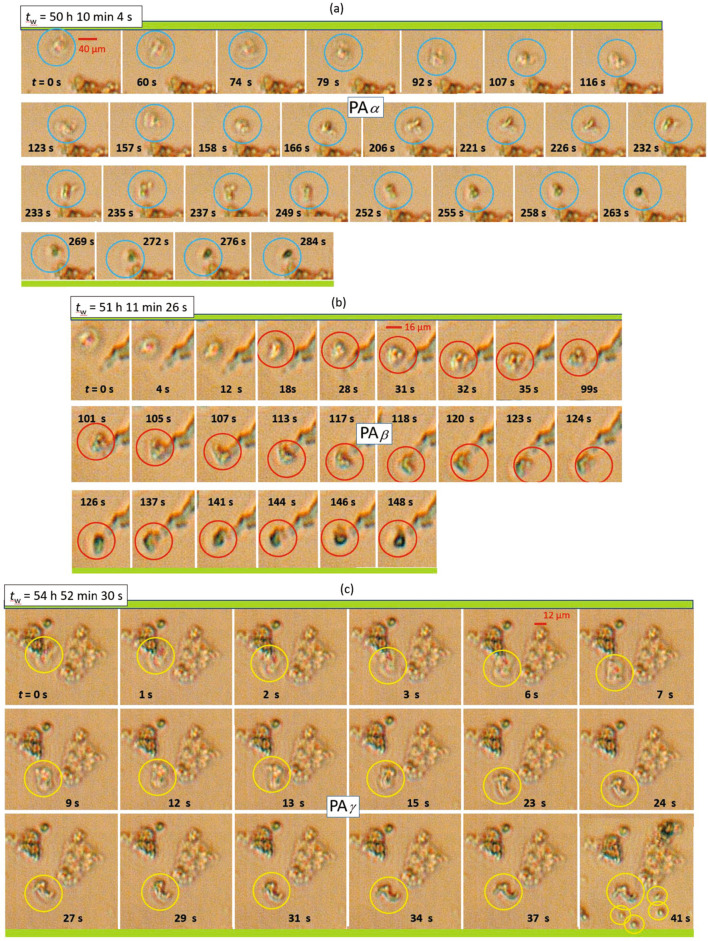
The response of various protein aggregates (PAs) formed at $c=1~\mathrm {mg/ml}$ to an electric field with $f=100~\mathrm {Hz}$ and $E=5~\mathrm {V/mm}$ as observed by time-resolved optical microscopy: (**a**) PA$_{\alpha }$, (**b**) PA$_{\beta }$, and (**c**) PA$_{\gamma }$. As a guide to the eye, individual PAs are indicated by circles. For each sample, the time between preparation and observation, $t_\text {w}$, is listed. For each morphology, the additional time span *t*, under an electric field, is provided, during which the aggregates are monitored.

Figure 2 shows the response of various amorphous aggregates formed at $c=1~\mathrm {mg/ml}$ to a weak AC sinusoidal field with a frequency $f=100~\mathrm {Hz}$ and a field strength $E=5~\mathrm {V/mm}$ (see also MOVIE 2). In Fig. 2a, PA$_{\alpha }$ shows three loops ($t=$ 60–74 $\mathrm {s}$) that merge together close to the region of interest $z_\text {ROI}$ and continue to form two neighboring tubes ($t= 116~\mathrm {s}$), one rather fixed in space, the other freely moving and rotating at $t= 284~\mathrm {s}$, until it hits the bottom plate. Figure 2b shows another aggregate, PA$_{\beta }$. It consists of at least three rapidly moving parts (cf. $t= 90~\mathrm {s}$) located close to an elongated aggregate. It continues to undergo local changes till $t= 124~\mathrm {s}$ and finally alters its orientations between $t= 126~\mathrm {s}$ and $146~\mathrm {s}$ until it completely stops at $t= 148~\mathrm {s}$. Moreover, a moving aggregate, PA$_{\gamma }$, is shown in Fig. 2c, that detaches from a PA cluster at $t= 7~\mathrm {s}$. Its full shape becomes visible at $t=13-15~\mathrm {s}$. It then keeps in motion till $t= 41~\mathrm {s}$ while the surrounding aggregates are steady on the bottom ITO glass, as checked by varying the *z* plane probed.

**Figure 3:**
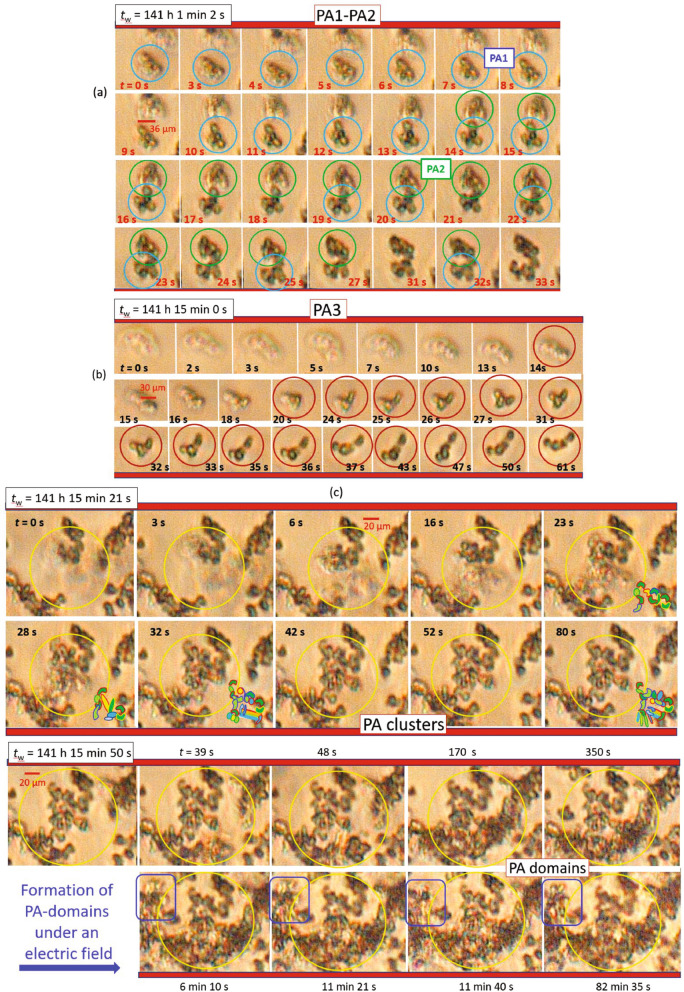
The response of large protein aggregates formed at $c=4~\mathrm {mg/ml}$ to an electric field with $f=100~\mathrm {Hz}$ and $E=5~\mathrm {V/mm}$ as observed by time-resolved optical microscopy: (**a**) PA$_1$ and PA$_2$, (**b**) PA$_3$, and (**c**) PA-clusters and the PA-domains. As a guide to the eye, individual PAs and regions of interest are indicated by circles and frames, respectively, in order to follow the field-induced temporal evolution of the morphologies. Cartoons (as insets) illustrate the configurational changes of the aggregate cluster.

**Figure 4:**
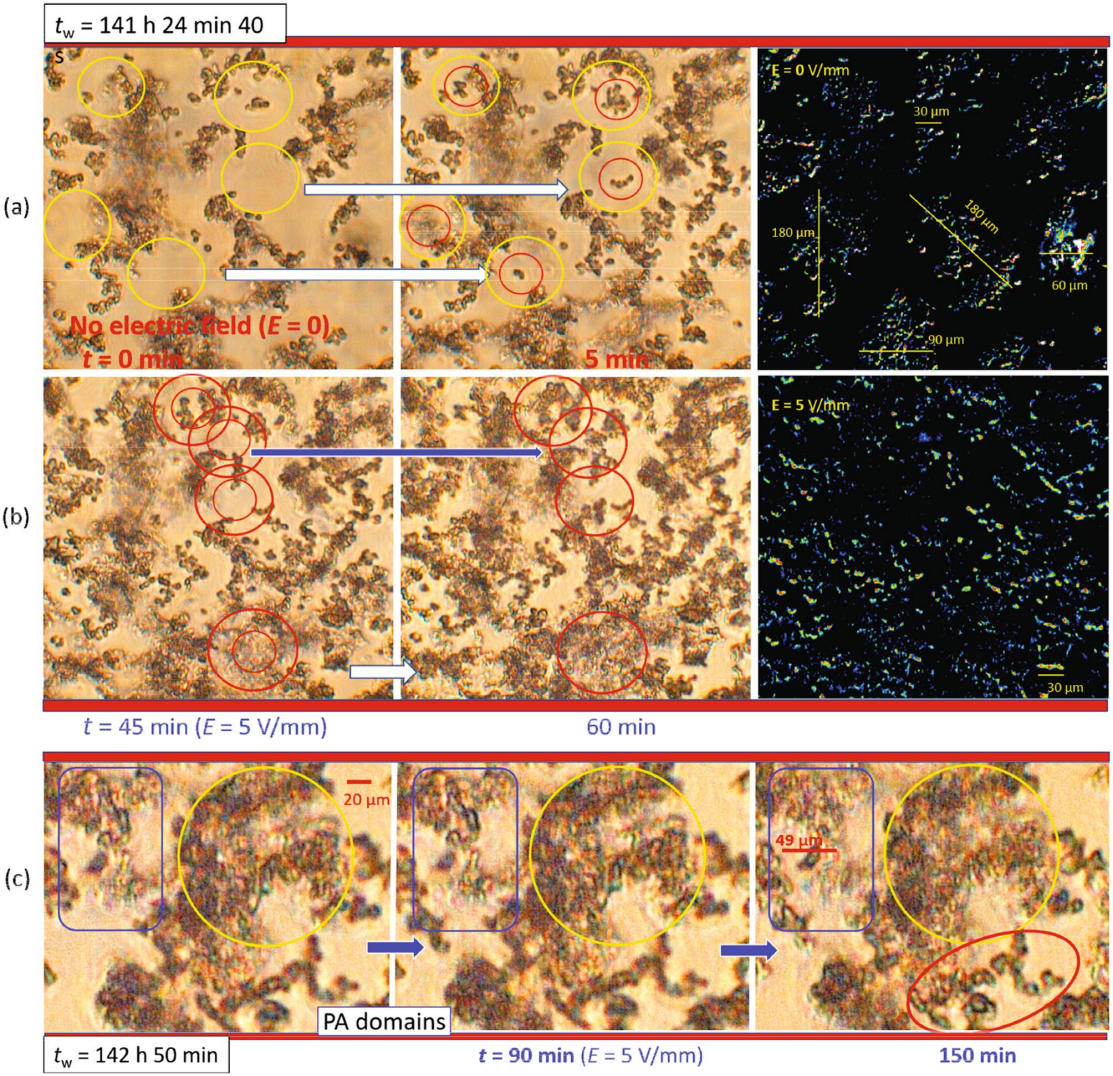
The response of large protein aggregates (PAs) formed at $c=4~\mathrm {mg/ml}$ to an electric field ($f=100~\mathrm {Hz}$, $E=5~\mathrm {V/mm}$): optical micrographs with at a larger field of view taken (**a**) without and (**b**) with an electric field applied. For clarity, the contrast-enhanced differences of the two images (**a**) without a field (taken at times 0 min and 5 min) and (**b**) with a field (taken at 45 min and 60 min) are displayed in the right panels. (**c**) Local rearrangement and disintegration of PA-domains observed after long-time exposure to an electric field. As a guide to the eye, different regions of interest are marked by circles and frames.

Figure 3 presents a collection of the characteristic features of PAs formed at $c=4~\mathrm {mg/ml}$ in the presence of a weak AC sinusoidal field ($f=100~\mathrm {Hz}$ and $E=5~\mathrm {V/mm}$): Fig. 3a shows the interaction of two PAs, PA$_1$ and PA$_2$: Initially, PA$_1$ moves, rearranges, and is clearly visible at $t= 8~\mathrm {s}$ while PA$_2$ is approaching. At $t=$ 14–18 $\mathrm {s}$, the shape of PA$_1$ is reformed while PA$_2$ is close, but has not yet fully developed its shape. At $t=19~\mathrm {s}$, PA$_2$ attains a configuration that is similar to that of PA$_1$, which undergoes a shape deformation at $t=21~\mathrm {s}$ and orientational changes in the following until it arrests. These observations illustrate that local rearrangements of PAs can occur under a low AC electric field. As shown in Fig. 3b, PA$_3$ slowly appears in the $z_\text {ROI}$-plane at $t= 20~\mathrm {s}$, rearranges its local configuration at $t= 31~\mathrm {s}$, followed by a stretching at $t=$ 15–18 $\mathrm {s}$ and a reorientation at $t=$ 20–31 $\mathrm {s}$. After a prolonged exposure to the electric field, the formation of clusters and domains of aggregates is observed (Fig. 3c). Yellow circles are used to highlight the differences in the observed optical morphologies. The morphology of more visible PA-clusters is clearly seen at $t=80~\mathrm {s}$.

Micrographs of this sample with a larger field of view are provided in Fig. 4 in the absence and presence of an electric field. When no field is applied, few PAs are obtained at $t={5}~\mathrm {min}$ (markey by red cricles in the middle panel of Fig. 4a). In contrast, in the presence of an electric field, not only more but also larger PAs are observed in a similar time span from $t=$ 45–60 $\mathrm {min}$ (again indicated by red circles in the left and middle panels of Fig. 4b). For a longer exposure time (not shown), local changes of clusters of aggregates are observed. The field effect on the reorientation, translational motion, and possible internal changes of the aggregates can be seen more clearly from the contrast-enhanced differences of the two images (shown as right panels in Fig. 4(a) without and (b) with a field). The difference images indicate that, without the field, large aggregates are still visible as a whole and hence only slightly change their configuration, while, with a field, smaller and more uniformly distributed changes occur due to more pronounced reorientation and translational motion of the aggregates. Moreover, Fig. 4c shows that the PA-domains do not only disintegrate locally, but also extend and locally rearrange ($t= 150~\mathrm {min}$). In addition, under the electric field, sharper optical interfaces of PAs are also seen at $t=150~\mathrm {min}$ (red ellipsoid in left panel of Fig. 4c).

### Field-induced deformation and reorientation of amorphous aggregates (formed at $c=0.4~\mathrm {mg/ml}$)

**Figure 5:**
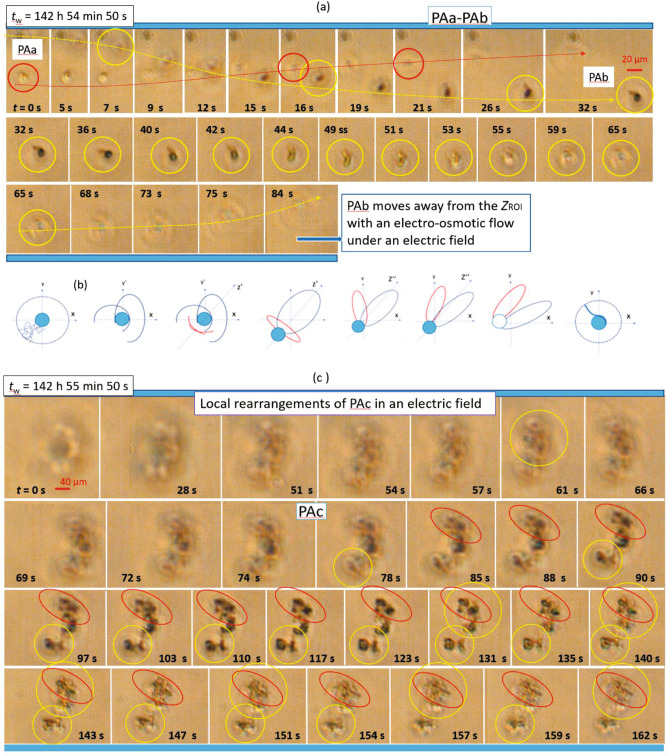
The response of protein aggregates (PAs) formed at $c=0.4~\mathrm {mg/ml}$ to an electric field with $f=100~\mathrm {Hz}$ and $E=5~\mathrm {V/mm}$ as observed by time-resolved optical microscopy: (**a**) PA$_\text {a}$ and PA$_\text {b}$, (**b**) a simplified illustration of various conformations of PA$_\text {b}$ approaching $z_\text {ROI}$ ($t=$ 5–26 $\mathrm {s}$), and (**c**) PA$_\text {c}$. As a guide to the eye, individual PAs are indicated by lines. The continuous configurational changes of the aggregates indicate local deformations of amorphous PA-clusters.

Figure 5 displays the characteristic features of PAs formed at $c=0.4~\mathrm {mg/ml}$ in the presence of a weak AC sinusoidal electric field ($f=100~\mathrm {Hz}$ and $E=5~\mathrm {V/mm}$). Figure 5a shows two protein aggregates, PA$_\text {a}$ and PA$_\text {b}$. Their trajectories are indicated by the red and yellow arrows, respectively. They appear to pass by each other at $t=$ 15–16 $\mathrm {s}$, but do not cluster together. This might be due to the presence of an electroosmotic flow with a noticeable effect at $t=32~\mathrm {s}$ (see the rightmost illustration in Fig. 5b) and at $t=65-84~\mathrm {s}$. It is conceivable that inter-aggregate interactions may differ, e.g., due to the hydrophobic effect, and that their effects appear to be more pronounced at $c=0.4~\mathrm {mg/ml}$ than in the systems with a higher total protein concentration, which hence typically contain larger, more compact and thus most probably more rigid aggregates. Accordingly, the dynamic mobility of the aggregates formed at $c=0.4~\mathrm {mg/ml}$ appears to be larger in the electric field, compared to those of samples with $c=1~\mathrm {mg/ml}$ and $c=4~\mathrm {mg/ml}$. This is further visualized in Fig. 5a: While PA$_\text {a}$ appears first in the region of interest, PA$_\text {b}$ only enters at $t=$ 32–36 $\mathrm {s}$ and undergoes configurational changes at $t=$ 40–44 $\mathrm {s}$, with in-plane connected and angled tubes at $t=$ 44–49 $\mathrm {s}$ and a centered loop at $t=51~\mathrm {s}$, eventually leaving $z_\text {ROI}$. Figure 5b schematically illustrates few steps of the configurational changes of PA$_\text {b}$.

Large-scale structures consisting of clusters of aggregates, like PA$_\text {c}$ in Fig. 5c, can also form at $c=0.4~\mathrm {mg/ml}$. In contrast to the steady behavior of protein aggregates at higher *c*, dynamical changes concomitant with local rearrangements of PA$_\text {c}$ are observed, including local mobility of the cluster, deformation and fragmentation of the cluster (pronounced at $t=$ 90–143 $\mathrm {s}$), and the formation of clusters with a stripe pattern at $t=162~\mathrm {s}$. (These differences can be seen clearly when comparing MOVIE 3 and MOVIE 1). The extent of local rearrangements might be related to the size of the aggregates and hence to the locally available space for rearrangements.

**Figure 6:**
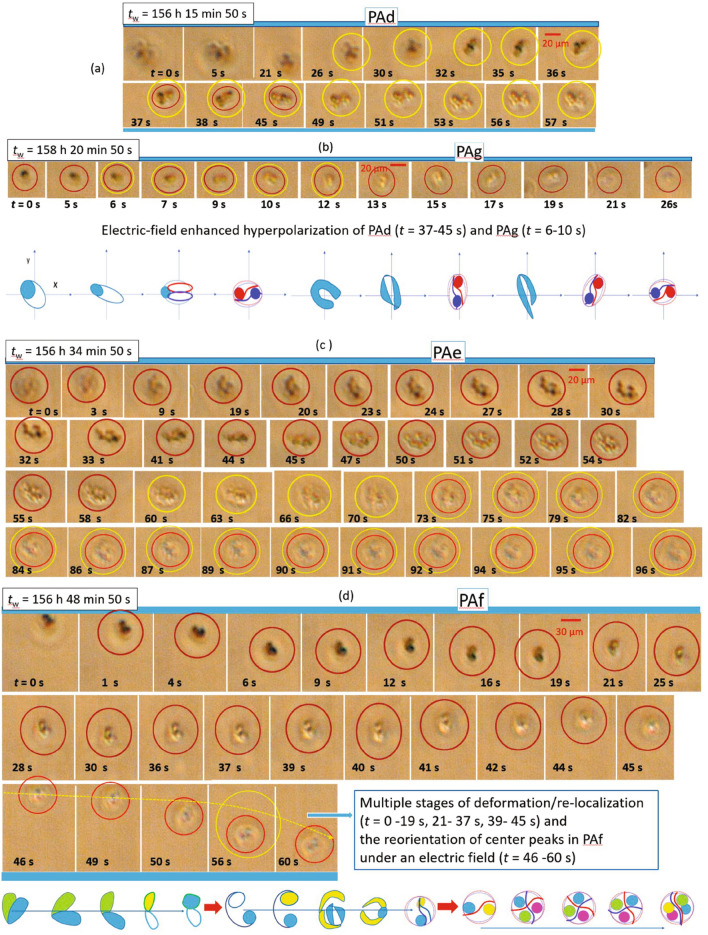
The response of protein aggregates (PAs) formed at $c=0.4~\mathrm {mg/ml}$ to an electric field with $f=100~\mathrm {Hz}$ and $E=5~\mathrm {V/mm}$ as observed by time-resolved optical microscopy (continued from Fig. 5): **a** PA$_\text {d}$, **b** PA$_\text {g}$ shown together with a simplified illustration of the hyper-polarization (indicated by red and navy colors) of the aggregate approaching $z_\text {ROI}$ and its reorientation ($t=$ 5–26 $\mathrm {s}$), (**c**) the aggregate cluster PA$_\text {e}$, and (**d**) the deformation and reorientation of PA$_\text {f}$ with the different steps illustrated by simplified cartoons shown below, for few steps of reorganization ($t =$ 1–19 $\mathrm {s}$, 25–45 $\mathrm {s}$, and 46–60 $\mathrm {s}$, from left to right). As a guide to the eye, individual PAs are indicated by circles.

The aggregates formed at $c=0.4~\mathrm {mg/ml}$ show distinct optically observed responses to the presence of a low AC electric field as illustrated in Fig. 6. PA$_\text {d}$ displays deformations (Fig. 6a, cp. $t=26~\mathrm {s}$ and $t=32~\mathrm {s}$) before settling and reorienting on the $z_\text {ROI}$ plane at $t=35~\mathrm {s}$. Even more drastic changes, like field-enhanced polarization (hyper-polarization), as observed in polarized morphologies, occur at $t=$ 37–45 $\mathrm {s}$, before PA$_\text {d}$ stops at $t=57~\mathrm {s}$.

Figure 6b also shows the reorientation of an aggregate with connected configurations, PA$_\text {g}$. Its reorientation is assisted by local hyper-polarization at $t=$ 6–10 $\mathrm {s}$, before the centered loop briefly occurs. A graphical representation of these changes is depicted in the lower part of Fig. 6b. Hyper-polarization is indicated by the separation of the protein aggregate into red and blue headed parts with different polarity. Even the reorientation of a larger PA-cluster, PA$_\text {e}$, (Fig. 6c) is observed at $t= 44~\mathrm {s}$, followed by an in-plane extension at $t=$ 45–55 $\mathrm {s}$ and changes of the orientations at $z_\text {ROI}$ at $t=$ 73–96 $\mathrm {s}$, before slowly leaving $z_\text {ROI}$. The field response of PA$_\text {f}$, in particular its reorientation, is clearly visible (Fig. 6d): two aggregates merge together, tilt at $t=$ 4–6 $\mathrm {s}$, undergo an in-plane realignment at $t=$ 12–16 $\mathrm {s}$ and changes of their lower part at $t=$ 16–19 $\mathrm {s}$ with two heads appearing at $t= 21~\mathrm {s}$ and a central loop at $t= 41~\mathrm {s}$ and after that leave $z_\text {ROI}$. Eventually, at $t=$ 46–60 $\mathrm {s}$, the cluster, whose center exhibits colored peaks, moves and rotates due to the presence of electro-osmotic flow. Some of consecutive steps of the changes of PA$_\text {f}$ are sketched schematically in the lower part of Fig. 6d, including deformations into a (1) stretched-out configuration, (2) a deformation localized in one of the PA units, leading to modulations, and (3) the slow rotation of the central PA, and moving away form the $z_\text {ROI}$ plane.

### Field-enhanced oscillations in DLS correlation functions of amorphous aggregate suspensions

**Figure 7:**
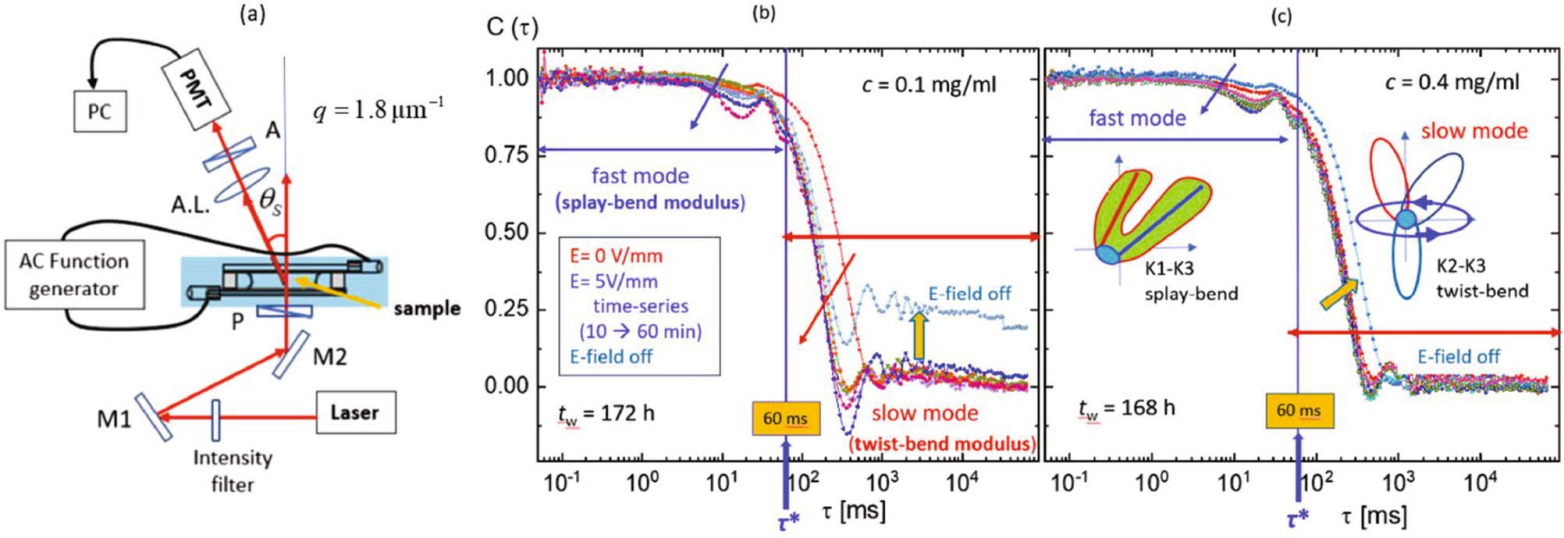
The collective microscopic dynamics of amorphous PA suspensions as investigated by in-situ electric-field small-angle dynamic light scattering (SAeDLS): (**a**) a simplified sketch of the light scattering set-up, operated at a fixed scattering angle $\theta _\text {s}$ corresponding to a magnitude of the scattering wavevector of $q= 1.8~\upmu \mathrm {m}^{-1}$, with mirrors (M1 and M2), a polarizer (P), an analyzer (A) and an achromatic lens (A.L., with a focal length of $75~\mathrm {mm}$); normalized intensity auto-correlation functions, $C(\tau )$, of aggregate solutions with (**b**) $c=0.1~\mathrm {mg/ml}$ and (**c**) $c=0.4~\mathrm {mg/ml}$ as a function of the correlation time $\tau$. Note that the characteristic correlation time, $\tau ^\star = 60~\mathrm {ms}$, is marked in order to distinguish the fast and the slow mode, indicated as $K_1$–$K_3$ (splay-bend) and $K_2$-$K_3$ (twist-bend) modulus, respectively.

Dynamic light scattering is employed to study the collective microscopic dynamics of amorphous PA suspensions. In particular, the field-induced deformation of the PAs is characterized in this way. The home-built small-angle electric-field dynamic light scattering (SAeDLS) set-up^50^ is sketched schematically in Fig. 7a. It is vertically aligned and allows to analyze the small-angle light scattering that is sensitive to the micron-sized protein aggregates. The effects of electric fields on the collective microscopic dynamics are investigated in situ for two samples, $c=0.1~\mathrm {mg/ml}$ and $c=0.4~\mathrm {mg/ml}$. The corresponding lag-time $\tau$ dependent, normalized intensity auto-correlation functions, $C(\tau )$, are presented in Fig. 7b,c, respectively.

Both Fig. 7b,c reveal local oscillation modulations in the whole correlation time range induced by an electric field with a frequency of $100~\mathrm {Hz}$ and an amplitude of $5~\mathrm {V/mm}$. The intensity auto-correlation functions $C(\tau )$ of PA suspensions show two relaxation modes: Thermal fluctuations of PAs and PA-clusters might be relevant for the fast and slow mode (less than $60~\mathrm {ms}$ and about 0.1–90 $\mathrm {s}$), resepctively. The slow mode is coupled with the low-frequency oscillations of the correlation function. A characteristic correlation time, $\tau ^\star =60~\mathrm {ms}$, is identified in Fig. 7b,c, distinguishing the two modes. They are commonly assigned to two types of elastic coupling moduli: one is the fast mode of the splay-bend ($K_1$–$K_3$) modulus and the other is the slow mode of the twist-bend ($K_2$–$K_3$) modulus in an elasticity theory of the suspensions^56–60^. The oscillations of the correlation function and hence the elastic deformations appear to be more pronounced at the lower *c*. Simple sketches of the moduli are shown as insets of Fig. 7c. When the electric field is applied, the local oscillation modulations due to the fast mode increases, while the slow mode decreases, as illustrated by the blue and red arrows, respectively. This will be discussed in more detail later (cf. Fig. 9; Table 1). After turning off the electric field (indicated by the thick yellow arrow), $C(\tau )$ decays to a non-zero long-time limit in Fig. 7b, while exhibiting a decay to zero in Fig. 7c. This is ascribed to the non-uniformity of the PA suspensions in terms of both polydispersity and dynamic heterogeneity at the lowest *c*. Also, for $c=0.4~\mathrm {mg/ml}$, only little variation of the field-induced oscillations is observed, further indicating different responses of the two samples.

To quantify the collective microscopic dynamics, in particular to examine the differences of the two samples, the correlation functions have been analyzed by model fits, considering the field-enhanced local oscillations. The intensity auto-correlation function is empirically described by the sum of a fast and a slow dynamical mode (denoted by subscripts f and s, respectively) via

1 $$\begin{aligned} C(\tau ) = \left\{ B + A_\text {f}\, \exp (-\Gamma _\text {f}\,\tau ) + A_\text {s}\, \exp (- \Gamma _\text {s}\,\tau ) \cos {[\varepsilon \, \sin (\Omega \,\tau +\phi )]} \right\} ^2\;. \end{aligned}$$

The amplitudes and relaxation rates are designated by *A* and $\Gamma$, respectively, and *B* is a background constant. The local oscillation modulation is accounted for by a slow-mode cosine term whose argument contains an amplitude $\varepsilon$ and a sine function with modulation frequency $\Omega$ and phase lag $\phi$. The physical significance of the parameters $\varepsilon$ and $\Omega$ is as follows. The amplitude $\varepsilon$ measures the degree of mechanical stiffness of the amorphous protein aggregates; the larger the amplitude the easier it is to deform the interior structure of the aggregates. The parameter $\Omega$ is the frequency with which the aggregates oscillate locally in response to the applied field. The origin of the modulation in low *c* samples could be interfacial optical contrast or solvent flow in the PA suspensions. The decay of the correlation function for polydisperse systems can often be described by a single stretched-exponential function. In our case, the oscillatory behavior due to the electric field should then be included by multiplying the stretched exponential with the cosine function as given in Eq. (1). Such an ansatz does not fit our correlation functions. We therefore chose to represent polydispersity by using two (non-stretched) exponential functions, where only the slow mode is multiplied by the cosine modulation term. It thus turns out that the larger aggregates in the dispersion, leading to the slow mode, are more susceptible to the electric field as compared to the smaller aggregates.

**Figure 8:**
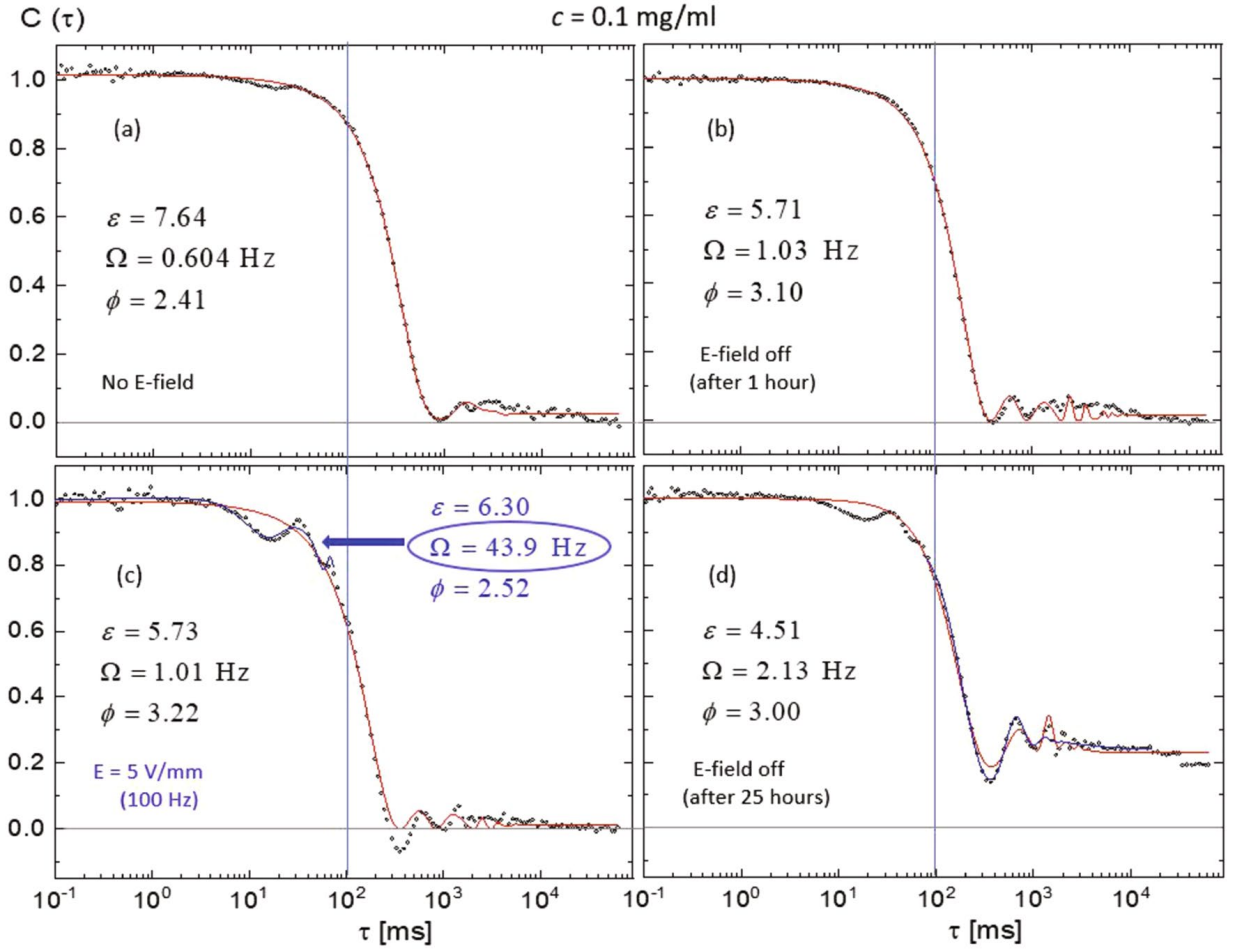
Exemplary data analysis of normalized intensity auto-correlation functions, $C(\tau )$, of aggregate solutions with $c=0.1~\mathrm {mg/ml}$ as a function of the correlation time $\tau$: data (symbols) and fits (solid lines): (**a**) without an electric field, (**b**) taken an hour after turning off the field, (**c**) in the presence of an electric field, and (**d**) taken 25 hours after turning off the field. The red line covers all data points, but the blue line in (**c**,**d**) is constrained to a limited data set as indicated. The parameters describing the local oscillation modulation are obtained from the fits: the local oscillation amplitude $\varepsilon$, the modulation frequency $\Omega$ and the phase lag $\phi$.

Exemplary fits (lines) to the data (symbols) are shown in Fig. 8 for $c=0.1~\mathrm {mg/ml}$ and various electric field conditions (as indicated). The resultant fit parameters are summarized in Table 1. Fits to the whole time range (red lines) and fits constrained either to the fast or slow mode (blue line in Fig. 8c,d) agree reasonably with the respective data, validating the approach of two different dynamical modes. Note that the field-induced oscillation peak of the fast mode is better fitted with an enhanced modulation frequency, $\Omega =43.9~\mathrm {Hz}$ compared to the fit to the whole time range ($\Omega =1.01~\mathrm {Hz}$). This is expected as the difference between the applied frequency and the response of protein aggregates is smaller and thereby the relaxation time contributes more in the fast-time window (below the characteristic time). Also, the response to releasing the field can depend on time for the lowest *c* sample: after a short time period, the overall relaxation is faster (Fig. 8b) than the one before applying the field (Fig. 8a). However, longer time after the release, $C(\tau )$ does not decorrelate completely, as explained above. Thus the two low *c* samples can be discerned by their different responses to the weak electric field.

**Figure 9:**
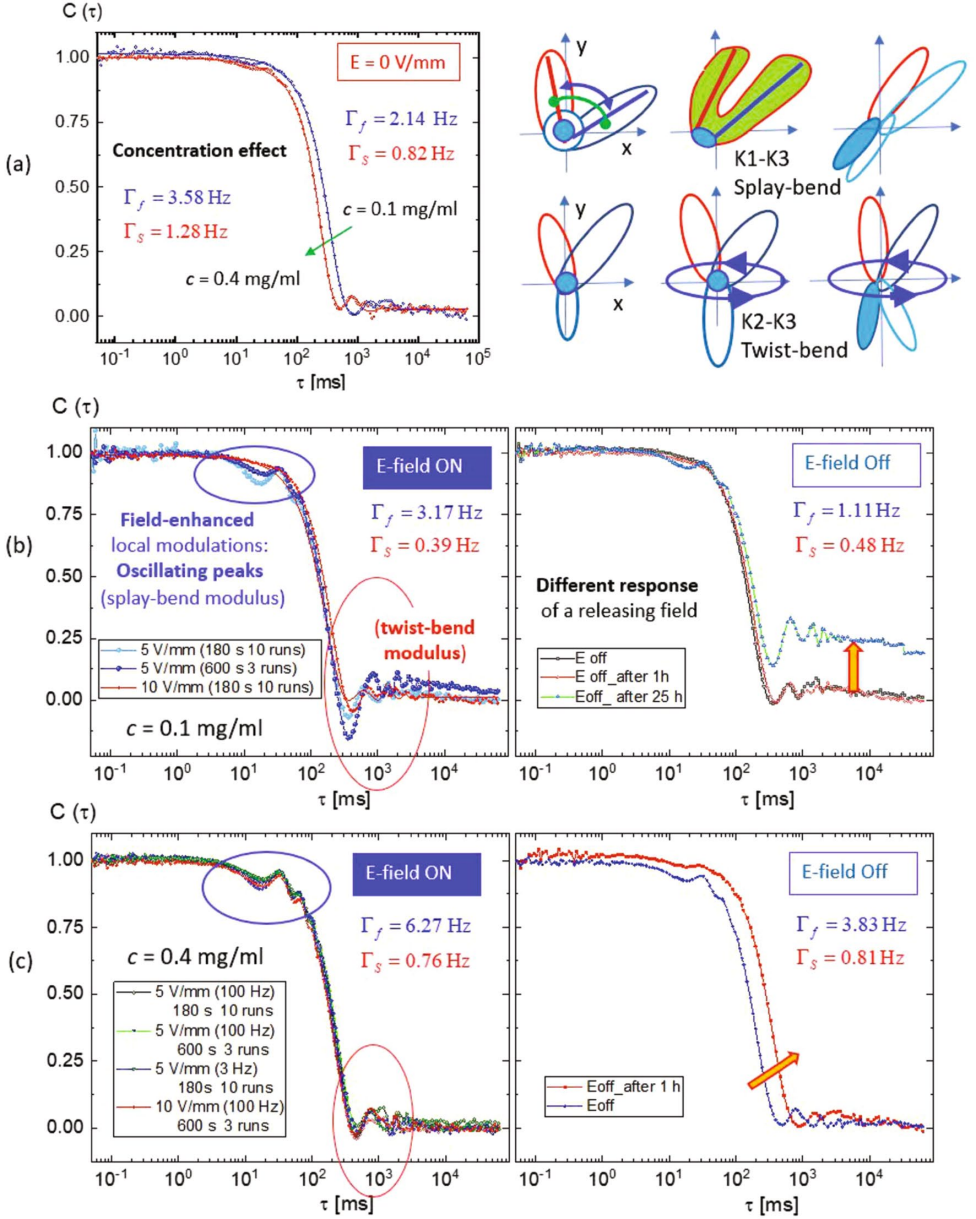
Normalized intensity auto-correlation functions, $C(\tau )$, of aggregate solutions as a function of the correlation time $\tau$: (**a**) in the absence of an electric field for $c=0.1~\mathrm {mg/ml}$ and $c=0.4~\mathrm {mg/ml}$, shown together with sketches of two possible elastic moduli, (**b**,**c**, right) in the presence of an electric field (with field parameters as indicated) as well as (**b**,**c**, left) after turning off the field for $c=0.1~\mathrm {mg/ml}$ (**b**) and $c=0.4~\mathrm {mg/ml}$ (**c**). The relaxation rates for the fast and slow mode, $\Gamma _\text {f}$ and $\Gamma _\text {s}$, result from fits of the local oscillations and are listed as insets. In the field (left panels of **b**,**c**), $C(\tau )$ exhibits field-enhanced local oscillation peaks. However, upon releasing the field (right panels of **b**,**c**), the two samples show different behavior, as denoted by the thick yellow arrows.

Figure 9 contains a detailed comparison of the field response of the samples $c=0.1~\mathrm {mg/ml}$ and $c=0.4~\mathrm {mg/ml}$. Based on model fits (shown as solid lines), the relaxation rates of the two dynamical modes, $\Gamma _\text {f}$ and $\Gamma _\text {s}$, are extracted. The respective values are listed as insets. In order to compare the concentration effect in the absence of an electric field, the normalized intensity-auto correlation functions for $c=0.1~\mathrm {mg/ml}$ and $c=0.4~\mathrm {mg/ml}$ are shown in Fig. 9a. Slightly shorter relaxation times are found for the higher *c*: $\Gamma _\text {f} = 2.14~\mathrm {Hz}$ compared to $\Gamma _\text {f}=3.58~\mathrm {Hz}$ in the fast mode as well as $\Gamma _\text {s} = 0.82~\mathrm {Hz}$ compared to $\Gamma _\text {s} = 1.28~\mathrm {Hz}$ in the slow mode. The local oscillation modulations are largely affected by the slow-mode coupling ($A_\text {s}/A_\text {f}\approx 0.8$), while the fast mode is compensated within the characteristic time $\tau ^\star = 60~\mathrm {ms}$ (cf. the fast-mode fits in Fig. 8c). The $K_1$–$K_3$ (splay-bend) modulus takes place faster at $\tau < \tau ^\star$. Then, the entire correlation function can be fitted and interpreted by the $K_2$–$K_3$ (twist-bend) modulus as dominant in the time range $\tau > \tau ^\star$. Simple illustrations of elastic deformations for the splay-bend ($K_1$–$K_3$) modulus and twist-bend ($K_2$–$K_3$) modulus are shown on the right panel of Fig. 9a.

In the presence of an electric field, the intensity-auto correlation functions with field-enhanced local oscillation modulations are shown in the left panels of Fig. 9b,c for $c=0.1~\mathrm {mg/ml}$ and $c=0.4~\mathrm {mg/ml}$, respectively. The resulting relaxation rates are also indicated. For $c=0.1~\mathrm {mg/ml}$, the difference between the relaxation rates of the two modes is enlarged if the field is applied (Fig. 9b): the rates of fast and slow mode increase and decrease to $\Gamma _\text {f} = 3.17~\mathrm {Hz}$ and $\Gamma _\text {s} = 0.39~\mathrm {Hz}$, respectively. However, after turning off the field, $\Gamma _\text {f}$ reduces to $1.11~\mathrm {Hz}$, while $\Gamma _\text {s}=0.48~\mathrm {Hz}$ almost remains constant. These rates are even lower than in the absence of a field (after an hour). In addition, correlation functions obtained from many short-time runs agree with those from long-time measurements, indicating ergodic behavior of the samples. By contrast, at $c=0.4~\mathrm {mg/ml}$ (Fig. 9c), the relaxation rates of the fast and slow mode are $\Gamma _\text {f} = 6.27~\mathrm {Hz}$ and $\Gamma _\text {s} = 0.76~\mathrm {Hz}$, respectively. This is twice faster than in the case of $c=0.1~\mathrm {mg/ml}$. When the field is turned off, $\Gamma _\text {f} = 3.83~\mathrm {Hz}$ and $\Gamma _\text {s} = 0.81~\mathrm {Hz}$. This might further explain the field-induced polarization as a consequence of effective modulations by two modes as also directly visualized (Fig. 6a).

**Table 1:** Parameters (with statistical uncertainties given in brackets) retrieved from model fits, according to Eq. (1), to the intensity auto-correlation function $C(\tau )$ for aggregate solutions formed at an inital protein concentration *c* for various field conditions (in the absence of an electric field, in the presence of an electric field with amplitude *E* and frequency $f=100~\mathrm {Hz}$ and after turning off the field) as indicated: the relaxation rates for the fast and slow mode, $\Gamma _\mathrm {f}$ and $\Gamma _\mathrm {s}$, respectively, the amplitude of the local oscillation modulation $\varepsilon$, the modulation frequency $\Omega$ and its phase lag $\phi$.

*c* $(\mathrm {mg/ml})$	*E* $(\mathrm {V/mm})$	$\Gamma _\mathrm {f}$ $(\mathrm {Hz})$	$\Gamma _\mathrm {s}$ $(\mathrm {Hz})$	$\varepsilon$ $(\mathrm {rad})$	$\Omega$ $(\mathrm {Hz})$	$\phi$ $(\mathrm {rad})$
0.1	0	2.14	0.82	7.64	0.62	2.41
0.1	5	3.17	0.39	4.5–9.1 (2.1)	0.61–2.13 (0.6)	2.4–3.2 (0.9)
0.1	off	1.11	0.48	5.5–5.7 (0.4)	1.03–1.09 (0.2)	3.06–3.10 (0.2)
0.4	0	3.58	1.28	10.4	0.92	1.79
0.4	5	6.27	0.76	9.1–12.6 (1.9)	0.92–1.26 (0.6)	1.9–5.6 (1.0)
0.4	off	1.11	0.48	7.64–9.15 (1.2)	1.13–6.04 (2.2)	1.98–2.41 (0.7)

The fit parameters are listed in Table 1. For $c=0.1~\mathrm {mg/ml}$ (Figs. 8, 9b), their ranges are $\varepsilon$ = 4.5–9.1 $\mathrm {rad}$, $\Omega =$ 0.61–2.13 $\mathrm {Hz}$, $\phi =$ 2.4–3.2 $\mathrm {rad}$, while for $c=0.4~\mathrm {mg/ml}$ (Fig. 9c) $\varepsilon =$ 9.1–12.6 $\mathrm {rad}$, $\Omega =$ 9.2–12.6 $\mathrm {Hz}$, and $\phi \,=$ 1.9–5.6 $\mathrm {rad}$. Overall, larger differences of two-mode relaxations occur when the field is applied: a wider range of the modulation frequency $\Omega$ for $c=0.1~\mathrm {mg/ml}$ and a broader range of phase lags $\phi$ for $c=0.4~\mathrm {mg/ml}$. The more pronounced mobility enhances the amplitude of the oscillations. However, after turning off the field, marginally different fit parameters are found compared to those in the presence and absence of the field. The subtle differences of the microscopic dynamics between the two samples are based on dynamic heterogeneities of the PAs and enhanced local oscillation modulations in two distinguishable modes. The parameters describing the local oscillation modulations are coupled to both the fast and the slow modes; the relaxation rates are found to vary by field-induced separation with increasing in the fast mode and decreasing in the slow mode. This further enhances the amplitudes of oscillation modulations of amorphous PA suspensions under an electric field and also explains the field-enhanced polarization (hyper-polarization) of PA$_\text {d}$ and PA$_\text {g}$ (Fig. 6a) as a consequence of modulations originated by two elastic moduli.

## Summary and discussion

Lysozyme is prone to form micron-sized amorphous aggregates if its disulfide bridges are reduced by DTT. The characteristic aggregate size depends on the total protein concentration *c*. Such amorphous PA suspensions have been used as a model system here. Their response to an external electric field is explored in situ by time-resolved optical microscopy and small-angle dynamic light scattering. If exposed to a low AC field, the PAs show characteristically different features, such as the formation of stable amorphous aggregates as well as clusters and domains of aggregates. Moreover, as inferred from microscopic observation, they can undergo field-induced deformation, polarization and reorientation. In particular, we observed: (1) clear effects on the microscopic morphology for aggregates formed at intermediate *c*; (2) field-induced local modulation and eventual disintegration of PA-domains after prolonged exposure to the field at high *c*; (3) some large PAs formed at low *c* floating in the electro-osmotic flow weakly balanced by the field (see MOVIE 3); (4) elastic deformations reflected in the collective microscopic dynamics of amorphous PA suspensions, in particular at low *c*; (5) large PAs formed at high *c* sediment faster than smaller PAs formed at low *c*. It is conceivable that the size and deformability and hence the field response of amorphous PAs, as reflected, e.g., in the characteristic correlation time, can be tuned not only by *c*, but also by the salt concentration, whereas the DTT concentration (if sufficiently large) has previously been found not to alter the aggregate properties^25^.

The mechanism of field-enhanced modulations of amorphous protein aggregates under a weak AC electric field can be explained as follows: The field enhances interactions between protein aggregates depending on their size and orientation. Ion fluxes are deflected depending on the aggregate size and induce volume polarization. Induced polarization charges can be large enough to induce inter-aggregate interactions, as similarly observed for spherical and rod-like colloids^61^. Even in the case of uncharged colloids, the field can lead to micro-structural changes via enhanced interaction energies. This is also the case if the surface charge density of the aggregate is small, so that the polarization charges increase due to accumulation of ions. If the surface charge density is very high (possibly for $c=$ 1–4 $\mathrm {mg/ml}$), a large portion of the charges is compensated by mobile condensed ions^62^.

To conclude, amorphous protein aggregates can be manipulated by a weak AC electric field. The field induces morphological changes of PAs, whose time evolution is resolved by optical microscopy, and enhanced local oscillations in the collective microscopic dynamics likely due to two different elastic moduli. Also, by releasing the field, the effect of electro-osmotic flow becomes stronger for very low *c*. The field affects not only the morphology of the amorphous protein aggregates, but also promotes the field-induced polarization of PAs, which can even lead to the formation of clusters of aggregates, followed by the disintegration of domains, leading to further elastic deformations of the amorphous PAs. This work might thus stimulate further general interest in steering protein aggregation by an electric field.

## Methods

### Sample preparations

Hen egg-white lysozyme (purified, salt-free) was purchased form Worthington Biochemicals (cat.no. LS002933) and used without further purification. Sodium phosphate, sodium chloride (NaCl), and dithiothreitol (DTT) were of reagent grade quality and used as received. Ultrapure water was produced by a water purification system. Stock solutions were prepared by dissolving lysozyme, NaCl and DTT in 20 mM sodium phosphate buffer at pH 7.0, respectively. Samples with dedicated protein concentrations ($c=0.1$, 0.4, 1, and $4~\mathrm {mg/ml}$) were prepared by mixing appropriate amounts of lysozyme, buffer, NaCl, and DTT stock solutions. NaCl was added to reach a total ionic strength of 0.1 M. Samples were stored at ambient conditions for at least two days during which virtually all disulfide-reduced lysozyme molecules assemble into highly flexible, micron-sized, amorphous aggregates, as previously observed under similar solution conditions^25^. Sample preparation and experiments are conducted at room temperature $(24\pm 2)\,^\circ \mathrm {C}$.

### In-situ electric cell and electrode polarization

The response of the sample to an externally applied weak AC electric field is probed in situ by time-resolved optical microscopy and dynamic light scattering. The home-built optically transparent in-situ electric cell is placed under an inverted optical microscope, as sketched schematically in Fig. 1b. It consists of two parallel ITO-coated float glasses (purchased from Präzisions Glas & Optik GmbH, CEC500S, with dimensions of $40\,\times \,70\,\mathrm {mm}^{2}$ and a thickness of $0.7\,\mathrm {mm}$), separated by $1~\mathrm {mm}$. The ITO layer has a high visible-light transmission ($90\%$) at $633~\mathrm {nm}$ and the coating thickness is 15 nm^50^. Before use, the glasses are thoroughly rinsed with a water–ethanol mixture to remove any organic residues. The sample (about $300\, \upmu \mathrm {l}$) is placed on the bottom plate inside a rectangular insulating PTFE film spacer (Armbrecht and Matthes GmbH, AR5038 and AR5038GP), followed by the upper electrode, placed on the lower plate and sealed with the black thin adhesive Teflon tapes. The ITO layers were then connected to a function generator (Avtech model AV-151G-B, frequency range 1–350 $\mathrm {kHz}$, maximum output voltage $200~\mathrm {V}$, load resistance $50~\mathrm {k}\Omega$) via electronic connection pins. A sinusoidally varying AC electric potential was applied to the electrodes. The counter rotating electric field streams close to the lower and upper ITO glasses are indicated in Fig. 1b.

Electrode polarization may occur near the surface of the ITO glasses in building up charges at frequencies below roughly $60\,\mathrm {Hz}$ for the skin depth of $10\%$ of the ITO glass thickness, as indicated by the asymmetric electric potential lines. Most measurements were done at $100\,\mathrm {Hz}$ and $5\,\mathrm {V/mm}$ and performed in a region of interest away from the electrode polarization regime, such that the system is largely affected by the bulk steady AC electric field perpetuation. However, ambient electro-osmotic flows were present in the low *c* samples, giving rise to rotational motions of PAs (see Figs. 5, 6, Movie 3). More details on the electrode polarization of an in-situ electric cell are provided in Ref.^61^.

### Inverted polarized optical microscopy

The experimental set-up for microscopic investigations is sketched schematically in Fig. 1b. The sample is mounted on an inverted polarized optical microscope (Carl Zeiss, Axiovert 40CFL model). Time-resolved depolarized optical micrographs ($1392\times 1044$ pixels) were collected by using a $40\times$ objective lens (Zeiss, LD Plan-Neoflular, $40\times /\,0.6$ Korr) and captured with a frame rate of 30 fps as continuous recordings (typically 6 runs, each lasting $40~\mathrm {min}$) with a high-resolution microscope camera (JENOPTIK, Progres GRYPHAX) in the live video mode. The field of view was a circular area with a typically diameter of 18–19 $\mathrm {mm}$. All the measurements were performed at the *z*-plane of interest, $z_\text {ROI}$, in which the PAs slowly sediment towards the lower ITO glass plate (see Fig. 1b).

### Small-angle electric-field dynamic light scattering (SAeDLS)

The set-up for a small-angle electric-field dynamic light scattering (SAeDLS) is sketched schematically in Fig. 7a. The beam of a He-Ne laser (JDS Uniphase Model 1145P) with a wavelength of $633~\mathrm {nm}$ is vertically aligned and passes through the sample. The scattered intensity is detected under a fixed, small angle, which corresponds to a magnitude of the scattering wavevector, $q= 1.8\,\upmu \mathrm {m}^{-1}$, chosen to match the length scale of small protein aggregates. An ALV-5000/EPP multiple tau digital real time correlator is used to quantify the intensity fluctuations related to the collective dynamics of aggregate suspensions. The samples are investigated under various electric field conditions $(3~\mathrm {Hz}$, $100~\mathrm {Hz}$, and 1–10 $\mathrm {kHz}$) and for duration times of 10–30 $\mathrm {min}$ in 3–10 runs. The set-up has already been used to study colloidal rods^61^ and further details on the instrumentation are given in Ref.^50^.

## Supplementary Information

Supplementary Video 1.

Supplementary Video 2.

Supplementary Video 3.
